# Shipping regulations lead to large reduction in cloud perturbations

**DOI:** 10.1073/pnas.2206885119

**Published:** 2022-10-03

**Authors:** Duncan Watson-Parris, Matthew W. Christensen, Angus Laurenson, Daniel Clewley, Edward Gryspeerdt, Philip Stier

**Affiliations:** ^a^Atmospheric, Oceanic and Planetary Physics, Department of Physics, University of Oxford, OX1 3PU Oxford, United Kingdom;; ^b^Atmospheric Science & Global Change Division, Pacific Northwest National Laboratory, Richland, WA 99354;; ^c^Centre for Geospatial Applications, Plymouth Marine Laboratory, Plymouth PL1 3DH, United Kingdom;; ^d^Space and Atmospheric Physics Group, Imperial College London, SW7 2AZ London, United Kingdom

**Keywords:** aerosol, climate, shipping, machine learning

## Abstract

Global shipping accounts for 13% of global emissions of SO_2_, which, once oxidized to sulfate aerosol, acts to cool the planet both directly by scattering sunlight and indirectly by increasing the albedo of clouds. This cooling due to sulfate aerosol offsets some of the warming effect of greenhouse gasses and is the largest uncertainty in determining the change in the Earth’s radiative balance by human activity. Ship tracks—the visible manifestation of the indirect of effect of ship emissions on clouds as quasi-linear features—have long provided an opportunity to quantify these effects. However, they have been arduous to catalog and typically studied only in particular regions for short periods of time. Using a machine-learning algorithm to automate their detection we catalog more than 1 million ship tracks to provide a global climatology. We use this to investigate the effect of stringent fuel regulations introduced by the International Maritime Organization in 2020 on their global prevalence since then, while accounting for the disruption in global commerce caused by COVID-19. We find a marked, but clearly nonlinear, decline in ship tracks globally: An 80% reduction in SO${}_{x}$ emissions causes only a 25% reduction in the number of tracks detected.

## Significance

Ship tracks have long been studied as a clear manifestation of broader anthropogenic aerosol effects, but typically only in specific regions or for relatively short periods of time. Now, with the help of a machine-learning algorithm we have detected all of the tracks across the world’s oceans over two decades—more than 1 million in total. This allows us to determine where tracks are more likely to form and the sensitivity of clouds to such perturbations. Crucially, we see a sharp reduction in tracks due to the more stringent ship emission regulations since 2020. This constitutes clear evidence of a global cloud response to environmental regulations despite no such change being observed in other cloud properties.

Ship emissions can occur in remote ocean environments, providing opportunities to study the effects of aerosol in isolation from other anthropogenic influences. The impact of these emissions on clouds by acting as cloud condensation nuclei and enhancing cloud droplet numbers (1) can manifest as a long, narrow region of enhanced cloud brightness. These ship tracks were noticed in some of the very first Earth observing missions (2) and have been extensively studied since (see ref. 3 for a recent review). Their compact structure allows for easy comparison with adjacent “clean” clouds, providing counterfactual evidence of nonlinear effects, which can otherwise be very challenging to measure (4).

## Ship Track Climatology

**Fig. 1.**
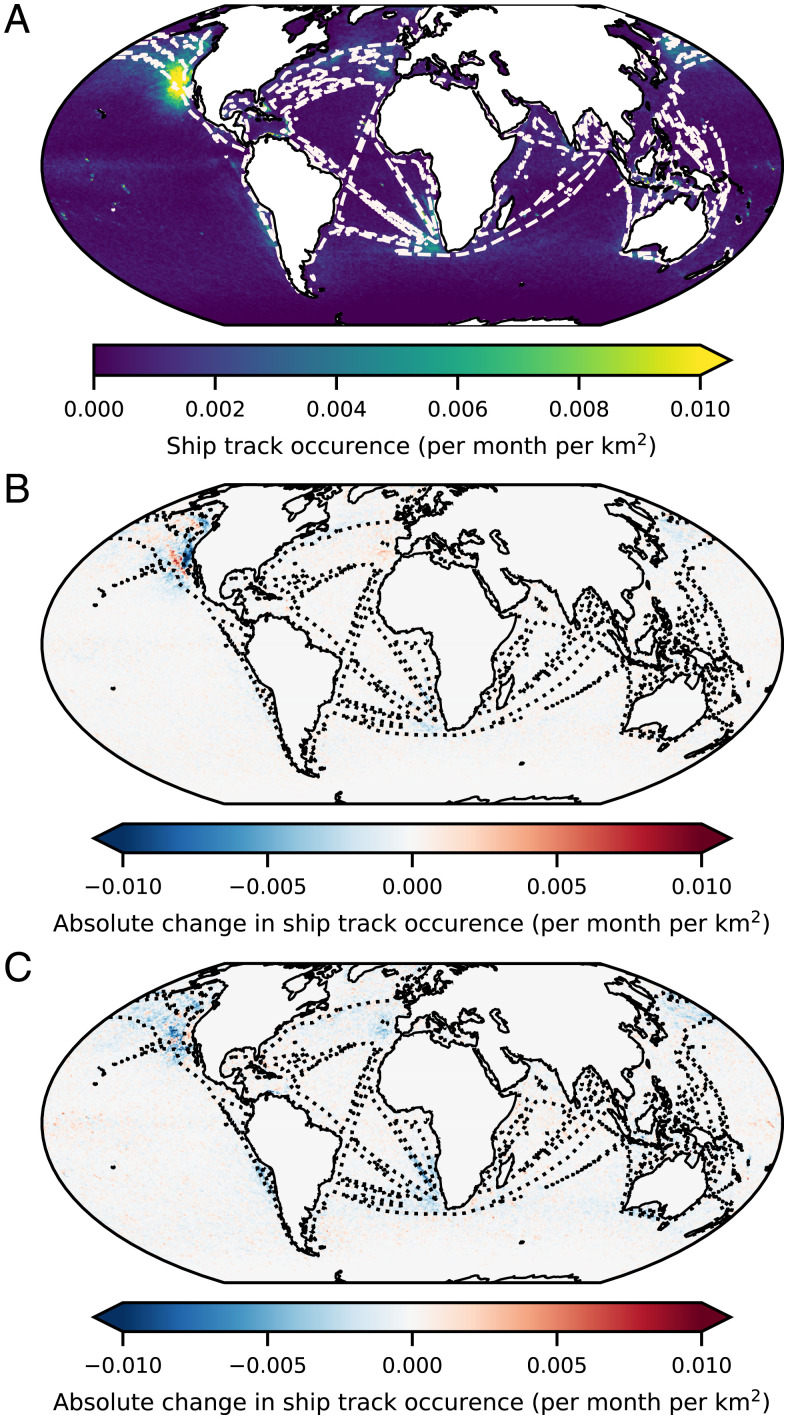
(*A*) The average monthly frequency of occurrence of ship tracks detected in Moderate Resolution Imaging Spectroradiometer (MODIS) Aqua imagery between 2002 and 2021 with a single contour level of average shipping emissions at 0.4 ng ⋅m^−2^ ⋅s^−1^ SO${}_{\text{x}}$ overlaid in white. (*B*) The absolute difference between the frequency of occurrence between 2002 to 2014 and 2015 to 2019 (inclusive), highlighting changes due to near-shore sulphur emission control area (SECA) emissions regulations. (*C*) The absolute difference between the frequency of occurrence between 2015 to 2019 and 2020 to 2021 (inclusive), highlighting changes due to IMO global shipping emissions regulations.

While the total radiative effect of detectable ship tracks is small, and the adjustments to the initial perturbation in droplet number are still contested (5), they nevertheless provide unique opportunities for experiments to quantify the effects of aerosol on clouds in general. While studies to date have focused on particular regions or cloud regimes and, at most, tens of thousands of examples, we use a machine-learning model trained on such hand-labeled datasets (*Materials and Methods*) to create a global database of more than 1 million ship tracks over a 20-y period, as shown in Fig. 1*A*.

This long-term, global view of ship track occurrence confirms the findings of previous studies that they are most prevalent in low and shallow marine stratocumulus (Sc) clouds found above the cold upwelling waters to the east of the major ocean basins. While the ship tracks are evenly dispersed over the Californian Sc deck, the prevailing meteorology in the Southeast Atlantic constrains these tracks very closely to the main shipping corridors (6). We also find significant numbers of tracks in other, more unexpected locations. There is a discernible increase in density along the shipping corridor along the South Indian Ocean and a high density along the Great Australian Bight. Not all of the detected tracks can be attributed to shipping, however. Local hotspots around Indonesia (shown in *SI Appendix*, Fig. S4) suggest these could be caused by the large number of volcanic sources in this region. Such tracks might provide valuable insights into these emissions when cloud cover would otherwise prevent remote-sensing estimates.

This database provides a unique opportunity to explore the spatial and temporal distribution of these features in different environmental conditions in response to a broad range of emissions. Indeed, the introduction by the International Maritime Organization (IMO) of stringent emissions limits in the emission control areas (ECAs) around the coast of North America and the North Sea, reducing the limit on sulfur (S) in fuel oil to 1% S (by mass) in 2010 and to 0.1% in 2015, and a global reduction on the limit from 3.5 to 0.5% after 1 January 2020 provide an opportunity to assess these sensitivities.* Unfortunately, at about the same time as the global emissions regulations came into force, the global COVID-19 pandemic took hold and disrupted global shipping (7), making a direct comparison with previous years challenging. By 2021, however, most shipping had returned to its prepandemic level (7) and a clearer picture of the impact of the regulatory changes is revealed.

**Fig. 2.**
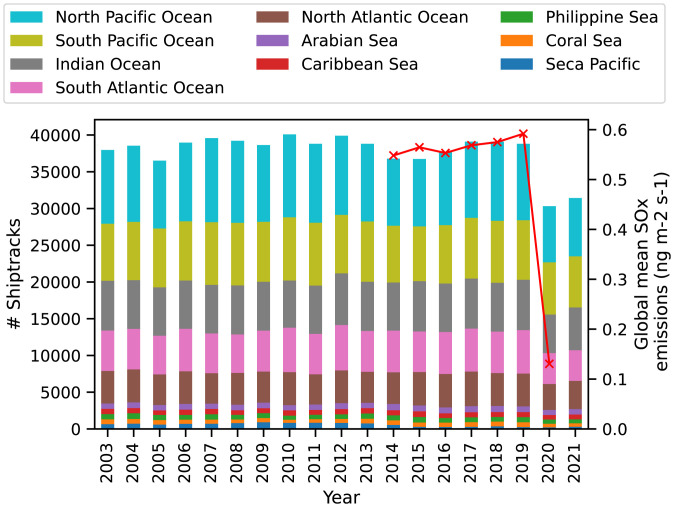
The total number of ship tracks by ocean region between 2003 and 2021 (inclusive), overlaid by the global mean shipping emissions of SO${}_{\text{x}}$ where available. Ocean region boundaries are shown in *SI Appendix*, Fig. S7.

The impact of these global events is distinctly seen in Fig. 2, which shows the total number of ship tracks detected across the 10 most common ocean basins over the last 19 y (discounting 2002, which had only partial coverage). While ~40,000 ship tracks formed every year until 2020, that year the number dropped to only 30,000—a 25% decline. The largest oceans tend to have the largest number of ship tracks and the change in 2020 occurs uniformly across all regions. As anticipated, there was a slight recovery in 2021 as the global shipping volume returned to normal levels, but well within the interannual variability of the previous years. As with other environmental indicators (8, 9), the effect of COVID-19 on the occurrence of ship tracks appears to be small compared to the natural variability and particularly compared with the regulatory changes. Indeed, the cumulative navigated miles in 2020 were ultimately only 3% lower than predicted (7, 10).

**Fig. 3.**
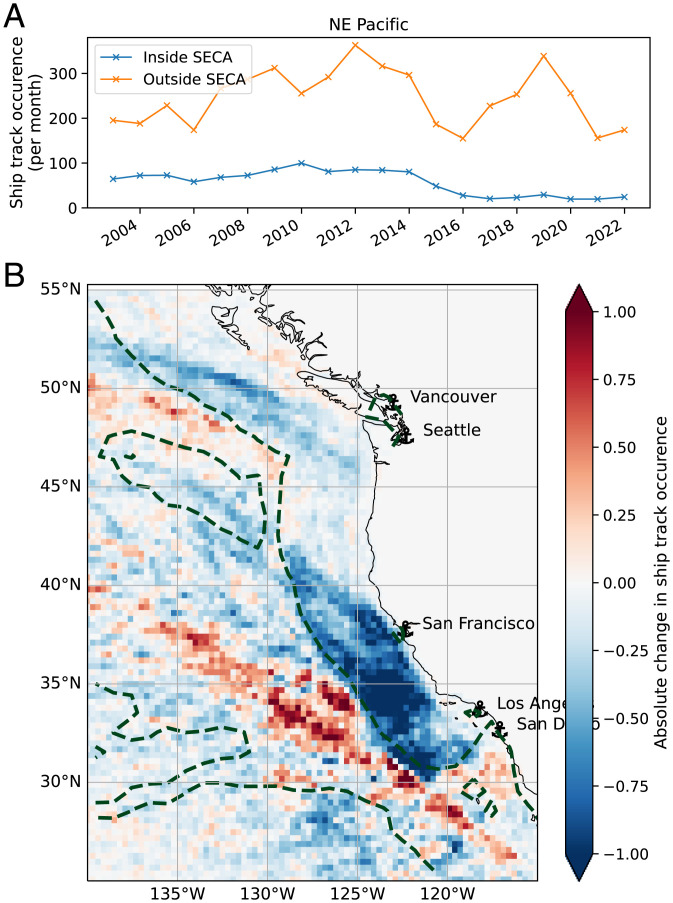
(*A* and *B*) Regional close-up of the difference due to ECA regulations changes (2002 to 2014 minus 2015 to 2019) off the coast of California. A time series of the ship track frequency of occurrence inside and outside the ECA region is also shown.

The spatial distribution of these changes is shown in Fig. 1 *B* and *C*, which shows the changes in ship track occurrence between 2002 to 2014 and 2015 to 2019 and between 2015 to 2019 and 2020 to 2021, highlighting the effect of regional and global regulatory changes, respectively. The changes seen in Fig. 1*B* clearly show the large reduction in ship tracks that occurred off the coast of California with the introduction of the 0.1% limit within the ECA around the North American coast, but no discernible change in the North Sea ECA, as has already been noted (11). A small reduction is seen in the Northwest Atlantic off the coast of Nova Scotia, but as few ship tracks are ever found here, the absolute change is negligible. There is a marked increase in ship tracks just outside the ECA in the North Pacific as shipping routes were changed to avoid the regulatory area between 2016 and 2019 (Fig. 3). There appears to be a small increase inside the ECA again in 2021 as the price differential between ECA and non-ECA routes is reduced. The changes due to IMO regulations are stark and much more uniform: There is a large reduction in ship track incidence everywhere they typically occur (see regional changes in *SI Appendix*, Fig. S3). This uniform reduction clearly shows the impact of, and general adherence to, the IMO regulations introduced in 2020.

## Sensitivity of Clouds to Ship Emissions

These clear reductions in ship track occurrence are in contrast to the broader changes in marine cloud droplet number that do not show any particular effect of the changes in regulations outside of the longer-term decline since around 2007 (*SI Appendix*, Fig. S5). Such large-scale changes have been attributed to total anthropogenic emissions changes over the period and also show a sublinear response (12). Even regionally though, the only discernible change occurs in the South Atlantic where the influence of continental aerosol sources may be less than in the South Pacific.

**Fig. 4.**
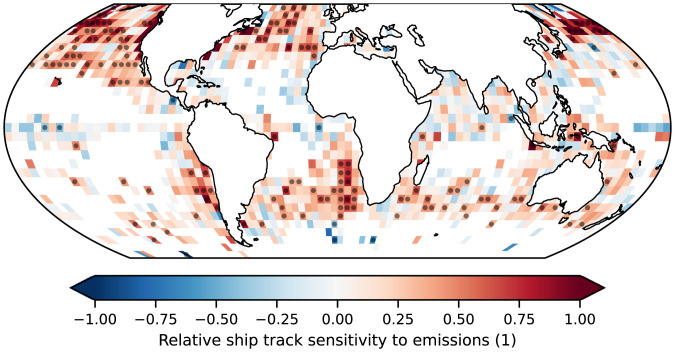
The sensitivity of relative change in ship track occurrence to relative change in shipping emissions of SO${}_{\text{x}}$ in 4^∘^ × 4^∘^ regions between 2014 and 2021 (inclusive) where ship track occurrence is greater than 0.1/mo. The stippling represents the rejection of the null hypothesis of no sensitivity at *P* < 0.05.

**Fig. 5.**
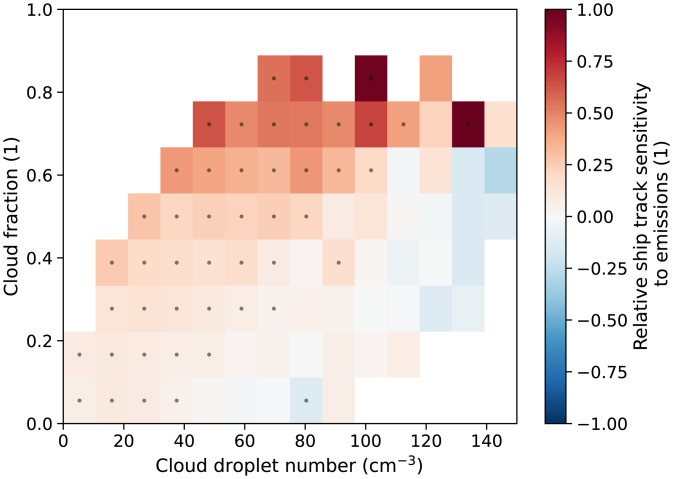
The relative change in ship track occurrence for a relative change in SO${}_{\text{x}}$ emissions as a function of average cloud fraction and background cloud droplet number in 4^∘^ × 4^∘^ regions.

By regressing the changes in ship track occurrence against the associated (large) changes in shipping emissions of SO${}_{\text{x}}$ we can determine the global sensitivity of clouds to these perturbations, as shown in Fig. 4. As expected, this sensitivity is positive everywhere and generally higher where ship tracks tend to be found since shipping covers a large portion of the ocean over multiyear timescales and the emissions reductions were uniform. Increased sensitivity can be seen in the extratropical shallow clouds, with the North Pacific and high cloud-fraction Sc particularly sensitive. Cloud fraction has been shown to play a leading role in determining the occurrence of ship tracks (11) and we find a similarly strong dependence, although there is also a (weaker) dependence on the background droplet number concentration: Cleaner clouds are more likely to produce ship tracks in response to ship emissions, as seen in Fig. 5.

While locally the relative sensitivity of ship track formation to emissions changes can be as large as 1.0, there is large spatial variability and the global change in the number of tracks is clearly sublinear: An 80% reduction in SO${}_{\text{x}}$ emissions causes only a 25% reduction in the number of tracks detected. Since the change in droplet number is known to respond logarithmically with increased condensation nuclei (13), this demonstrates how far from their preindustrial conditions the shipping corridors are, even after such a large reduction in emissions. It also highlights the difficulty faced by proposed marine cloud brightening efforts due to the diminishing returns on injected aerosol.

## Discussion

Ship tracks can generally be discerned (either manually or automatically) only in homogenous cloud fields but, although hard to detect, cloud perturbations in inhomogeneous clouds such as broken cumulus can exist (14) and have recently been shown to have distinct and important liquid water path responses (15). Future work will combine these approaches to better determine the radiative forcing induced by shipping and the degree to which cloud perturbations are saturated by present anthropogenic emissions. Such an approach would also allow a determination of the sensitivity of this, and other ship track detection studies, to the brightness and linearity of the tracks.

By detecting and analyzing more than 1 million ship tracks over two decades we have been able to unambiguously demonstrate the response of anthropogenic changes in clouds to changing emissions, despite a negligible response in other background cloud properties over the period. This unique dataset highlights the impact of the successful implementation of the global aerosol emissions control regulations on the climate system and the limited effect of the COVID-19 pandemic. Combining the vast amount of Earth observing data now available with modern machine-learning techniques provides additional ways to assess global emission perturbations and will allow governments and international regulatory bodies to monitor the compliance to, and climate effects of, much needed emissions reductions schemes.

## Materials and Methods

### Training Data.

The model input comprises MODIS “day microphysics” composites, inspired by ref. 16 and constructed (using SatPy) from channels 1, 20, and 32 (corresponding to wavelengths of 645 nm, 3.75 µm, and 12.5 µm, respectively). This composite was designed to provide information in the visible (toward the middle of the solar spectrum), the near infrared (which provides information about the cloud droplet size), and the infrared (which allows discrimination of cloud liquid and ice). Histogram equalization was applied to scale each channel prior to training and inference. The original 1,350 × 2,030-pixel (px) images were bilinearly interpolated to 1,344 × 2,240 px and then split into 15,448 × 448-px images to be as large as possible while enabling a batch size of 8 during training and maintaining the full 1-km resolution. The training data were provided in the form of 4,500 hand-logged tracks marking the head and each turning point along the track (4, 11, 17, 18). These points were connected by straight lines of width 10 px, approximating the average ship track width of 9 km (19), and converted into 4,320,448 × 448-px bitmasks for use in training the model (20). An example image and the corresponding hand-logged data are shown in *SI Appendix*, Fig. S1.

### Ship Track Detection Model.

The ship track detection model (21) is a standard neural-network–based segmentation model with a UNet architecture (22), a resnet-152 backbone (23) pretrained on the 2012 ImageNet Large Scale Visual Recognition Challenge ImageNet dataset (24), and sigmoid activation on the final layer. We train using Adam optimization (25) with a learning rate of 0.01 and a batch size of 16 over 100 epochs on two NVIDIA Tesla V100s using tensorflow-distributed training. The learning rate is reduced by a factor of 0.2 if the validation loss is deemed to have plateaued over the last five epochs. We use a binary cross-entropy Jaccard loss and find this performs slightly better than a focal loss, while both perform significantly better than a standard binary cross-entropy due to the large class imbalance in the images. We found that introducing an augmentation step whereby each image is randomly flipped or rotated 90^∘^ also improves training slightly.

*SI Appendix*, Fig. S1 shows example model predictions alongside the (held-back) test masks. The model does well in a wide range of challenging scenes. As with traditional ship track studies, the algorithm we use is sensitive to both shape and microphysical perturbation, so older tracks with diminished Nd perturbations will be unlikely to be detected.

We briefly highlight a few of the key differences between this architecture and the only other published model (26), henceforth TY2019. Our model utilizes much larger image tiles than TY2019 (448-px square as opposed to 64-px square), thus allowing the model to learn more context, avoid artificial splitting of tracks, and therefor detect longer tracks. Indeed, we find somewhat fewer tracks (37,947 compared to 70,338) when searching the same region off the coast of California (180 to 100${}^{{}^{\circ}}$W, 0 to 60${}^{{}^{\circ}}$N) during 2010 as TY2019.

Our model is evaluated using the Jaccard index, or intersection over union (IOU): $J\left(A,B\right)=\frac{\left|A\cap B\right|}{\left|A\cup B\right|}$, where *A* is the binary target mask and *B* the model predicted mask. The test IOU of our model is lower than the reported value in TY2019 (53% compared to 91%) and this is partly due to the larger tiles, which makes the features relatively smaller and high IOUs harder to achieve. This could also be due to the larger range of training and, hence, test regions we used. We used three channels, including the two that were used in TY2019 to calculate the brightness temperature difference, which we hoped would allow the model to generalize better to different cloud regimes (TY2019 was used only in shallow stratocumulus clouds off the coast of California) and allow our model to work during the daytime when cloud microphysical retrievals are also available. The resulting data for TY2019 are not publicly available but their figure 1 seems to show indications of false positives that we try to avoid with the Jaccard loss and by including a small proportion of example images with no ship tracks (10%).

While many model architectures and training structures were explored during development, we highlight three distinct cases in *SI Appendix*, Table S1. The effect of augmentation is clearly seen with a reduction in IOU of nearly 20% when it is not used. We also trained a feature pyramid network (FPN) that uses a quite different architecture and has been shown to be skillful in image segmentation tasks (27). This performed reasonably well in terms of IOU (and comparably to the ResUNet) but produced feature masks that were somewhat more uncertain and less useful for our task of detecting specific tracks as seen in *SI Appendix*, Fig. S2. Given the importance of the number of detected ship tracks in a given tile, we also compared this metric in the test data and found a small overestimate in all models compared to the hand-logged tracks, with a SD of around 10%. Reassuringly, the best model in terms of IOU also performs best in the number of detected tracks.

Because the training data were collected from previous studies, they are somewhat biased toward cloud regimes and meteorological conditions in which ship tracks are already known to be prevalent. To assess the skill of the model in unseen regions we randomly select a scene from the Indian Ocean within which we find many tracks but that has not been extensively studied and for which no training examples are used. As shown in *SI Appendix*, Fig. S6, the algorithm robustly detects the six tracks in this complex scene.

We make our model as well as our training and test data public in the hope to encourage extension and reuse but also for easy comparison between different models and hope others will do the same.

### Analysis.

Inference was carried out over all available “MYD021KM” calibrated radiance files from the MODIS instrument on Aqua between 2002 and 2021 inclusive, totaling more than 250 TB of data (28). To achieve this, preprocessing, inference, and postprocessing were performed on MAGEO (Massive GPU for Earth Observation), a cluster of five NVIDIA DTG-1 max-Q nodes, operated as part of NEODAAS (Natural Environment Research Council [NERC] Earth Observation Data Analysis and AI Service), which provided a total of 40 Tesla V100 GPUs (200,000 CUDA cores), 400 CPU cores, and 2.5 TB of RAM. Ship track polygons were determined from contours of 50 and 80% confidence in each inferred mask and the resulting geolocated objects saved in a geographic information system database (29). While the model was found to generalize well to unseen regions of the globe, a marked increase in false positives was found in cold frontal clouds near each pole and over very bright desert surfaces. The average 12.5-µm brightness temperature was determined for each track and those found to be less than 273 K or over land were filtered out of the analysis set. While the full unfiltered dataset is available, all results and figures quoted in the text refer the filtered dataset. Ocean regions are determined using the centroid of each ship track and the Natural Earth ocean basin polygons shown in *SI Appendix*, Fig. S7. The maps of ship track density presented in Fig. 1 were determined by counting the number of shiptrack polygons that intersect the centroid of each 0.1^∘^ gridbox each month.

Ship-borne SO${}_{\text{x}}$ emissions data are obtained from the monthly CAMS-GLOB-SHIP v3.1 product at 0.1^∘^ resolution (30). The sensitivity of ship track occurrence to SO${}_{\text{x}}$ emissions is calculated using these data after taking the mean over 40 × 40 grid cells to upscale the resolution to 4^∘^. To determine the sensitivity of ship track formation to emissions as a function of environmental controls (Fig. 5) we use the mean single-layer retrieved liquid cloud fraction from the monthly MODIS level 3 product (MYD08_L3). The background droplet number concentration is calculated using the condensation rate temperature corrected adiabatic approximation (31, 32).

## Supplementary Material

Supplementary File

## Data Availability

Machine learning training data, inference output and all analysis data have been made available as follows:• The raw machine learning output, including segmentation masks: 10.5285/0d88dc06fd514e8199cdd653f00a7be0 (28) • The derived data: 10.5281/zenodo.7038703 (29) • Machine learning training data: 10.5281/zenodo.7038715 (20) • The machine learning algorithm and associated code: 10.5281/zenodo.7038855 (21).

## Footnotes

^*^https://www.imo.org/en/MediaCentre/HotTopics/Pages/Sulphur-2020.aspx, last accessed 31 January 2022.
