# Supplementary material for: Shipping regulations lead to large reduction in cloud perturbations
Source: Proc Natl Acad Sci U S A. 2022 Oct 3;119(41):e2206885119. doi: 10.1073/pnas.2206885119 (PMC9565328; doi:10.1073/pnas.2206885119)
Supplement: Supplementary File [file pnas.2206885119.sapp.pdf]

PNAS
www.pnas.org

Supplementary Information for

Shipping regulations lead to large reduction in cloud perturbations

Duncan Watson-Parris, Matthew Christensen, Angus Laurenson, Daniel Clewley, Ed Gryspeerdt and Philip Stier

Duncan Watson-Parris.
E-mail: duncan.watson-parris@physics.ox.ac.uk

This PDF file includes:

Figs. S1 to S7
Table S1

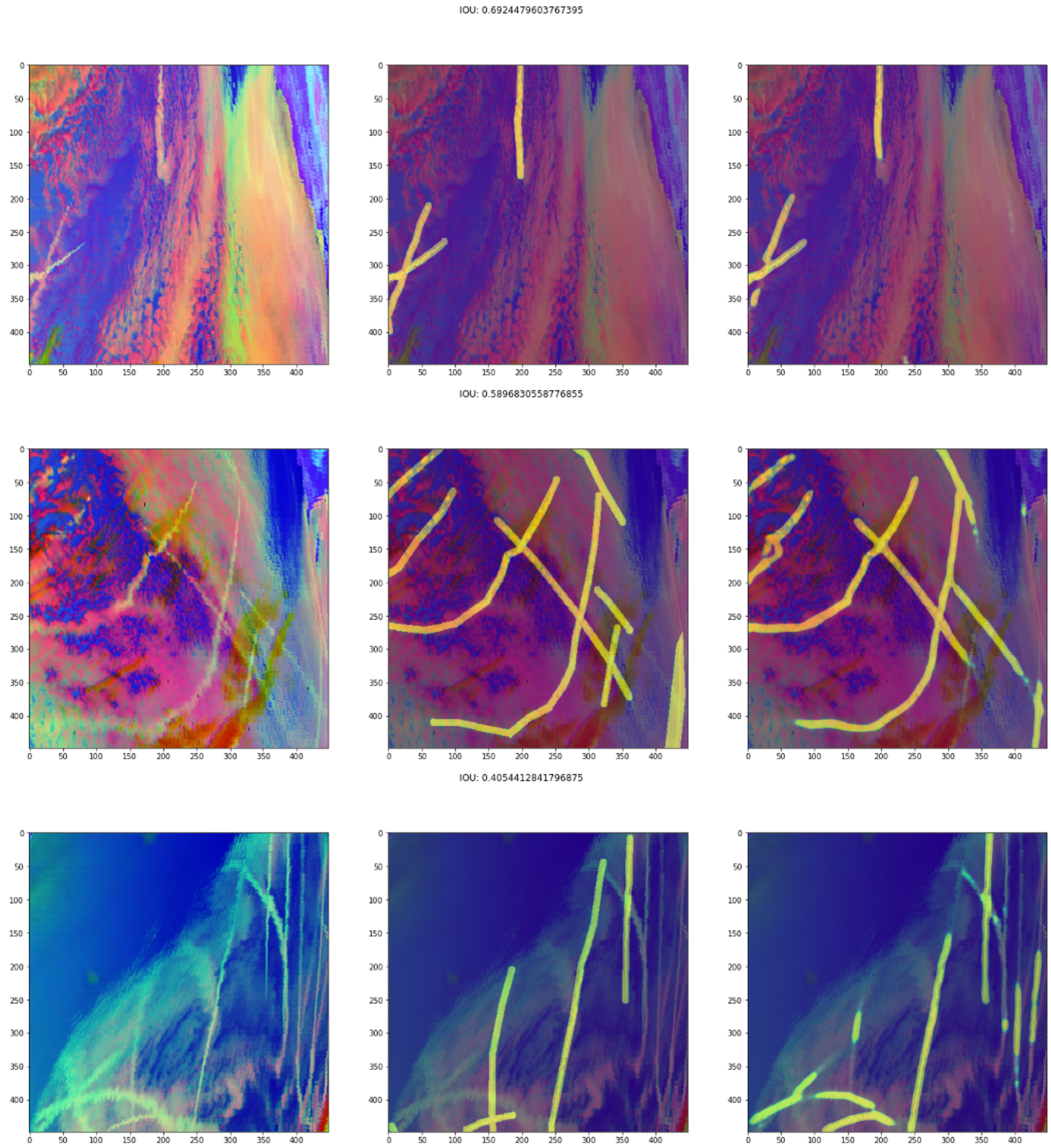

Fig. S1. Example images (a); hand labelled test mask which were not used during testing (b); and ML model prediction (c). The intersection-over-union (IOU) for each set is indicated in the corresponding titles.

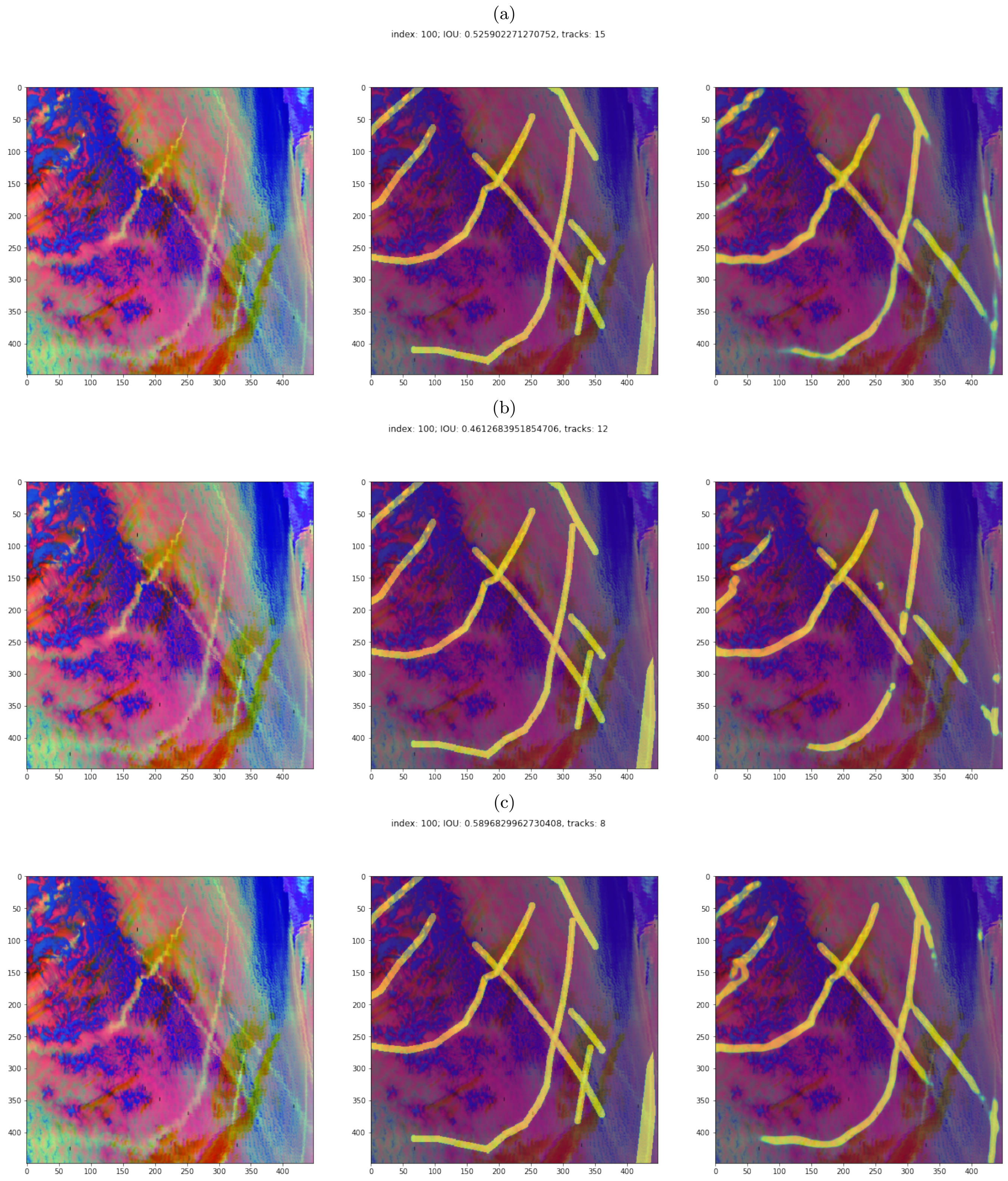

Fig. S2. Further examples of Figure S1b for the FPN model (a); and the final ResUNet without (b) and with (c) augmentation. Note, Figure c is the same as Figure S1b, and is included for easier comparison with the other models.

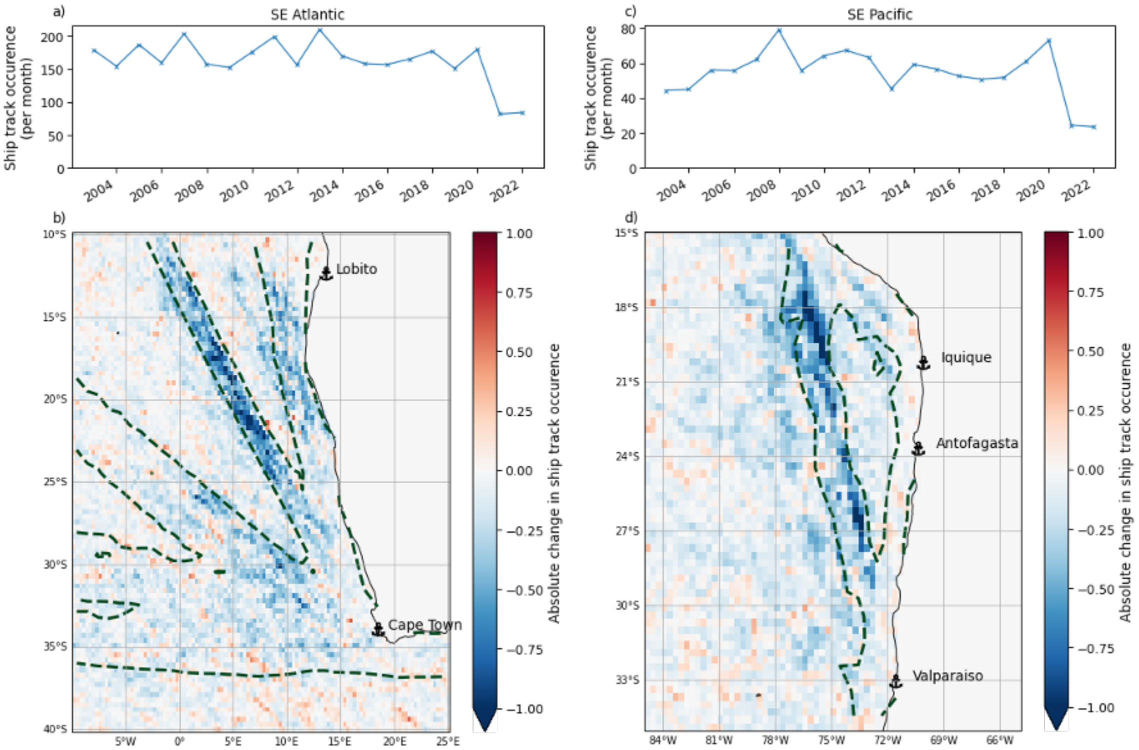

Fig. S3. Regional close-ups of the difference due to global IMO regulations changes (2015-2019 minus 2020-2021): (b) SE Atlantic, (d) SE Pacific. Time-series of the ship track frequency of occurrence over each region is also shown in (a) and (c) respectively.

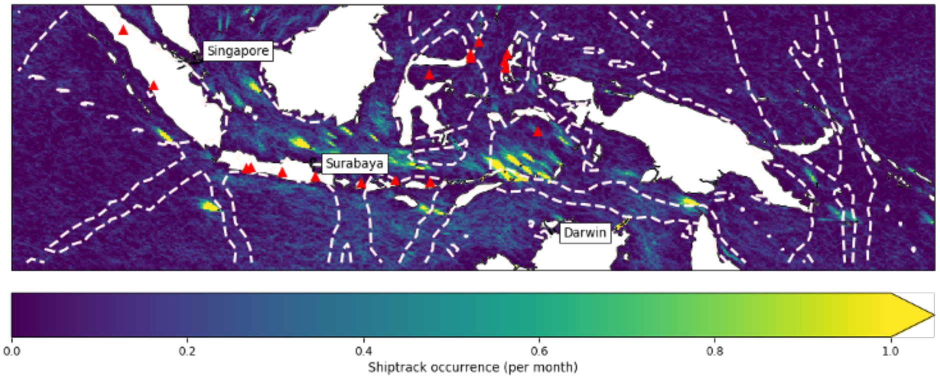

Fig. S4. Automatically detected cloud tracks around Indonesia, potentially due to volcanic rather than shipping sources. Known volcanic sources are indicated with red triangles.

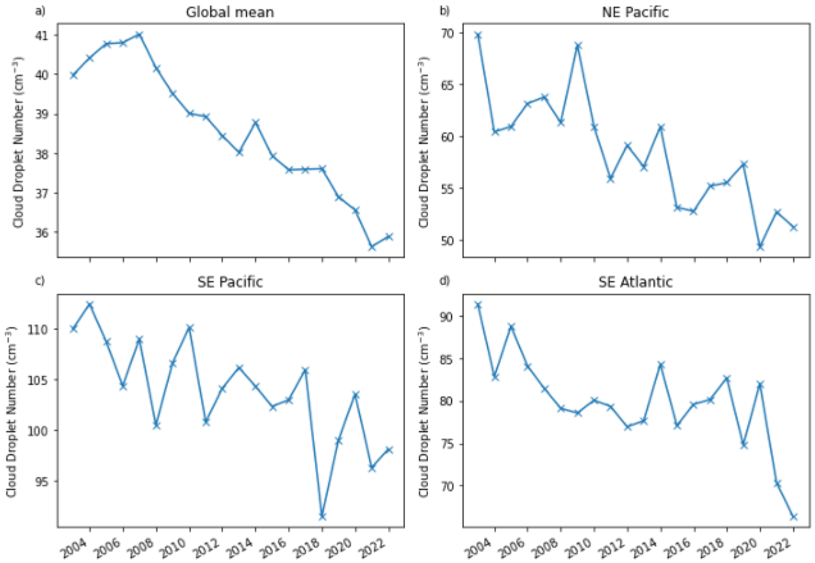

Fig. S5. Mean MODIS retrieved cloud droplet number concentrations (a) globally; (b) in the North-East Pacific; (c) the South-East Pacific; and (d) the South-East Atlantic.

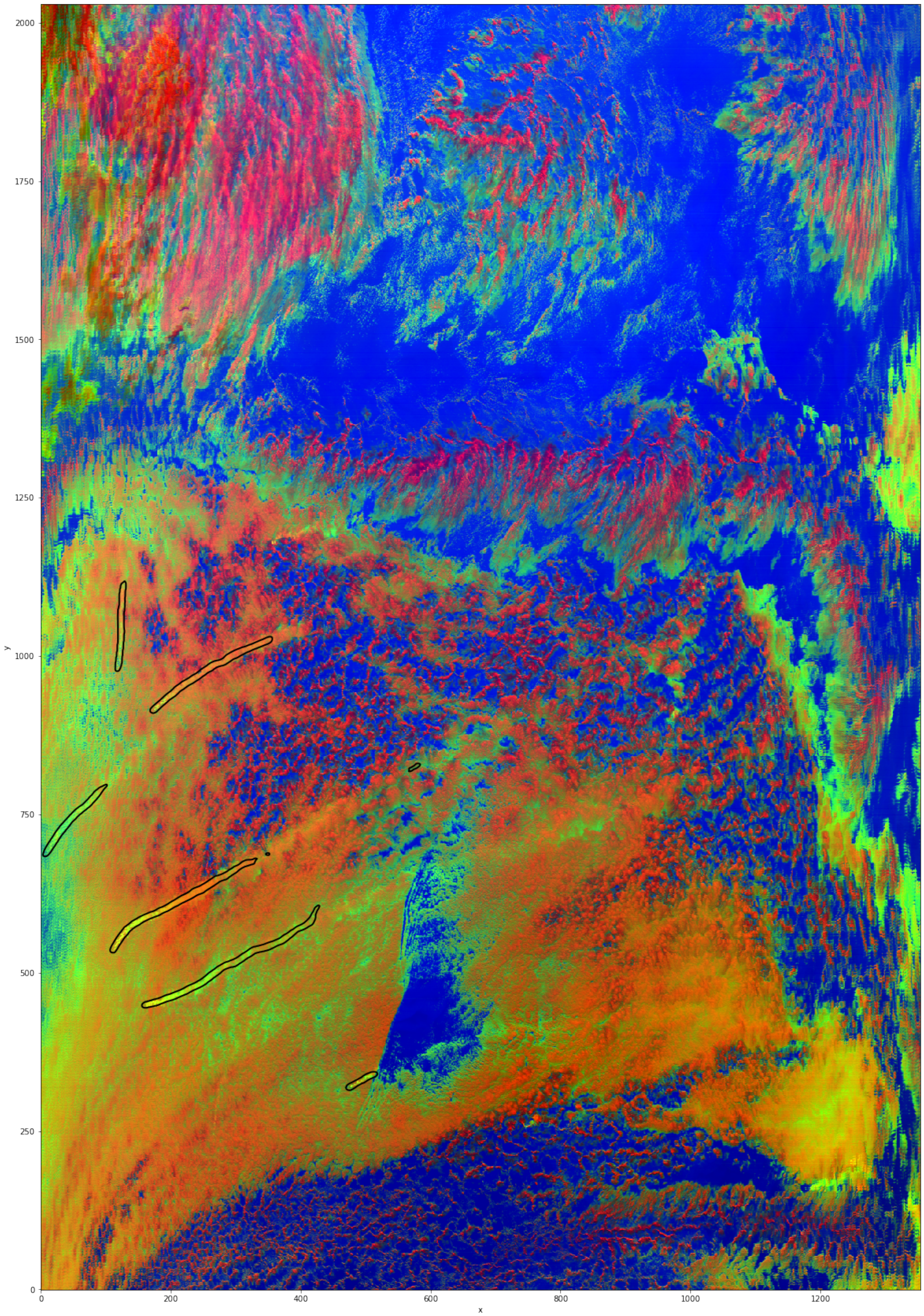

Fig. S6. Example full-resolution MODIS image taken at 08:55UTC on the 6th July 2002 over the southern Indian Ocean. Shiptracks detected using the described ML algorithm are highlighted in black.

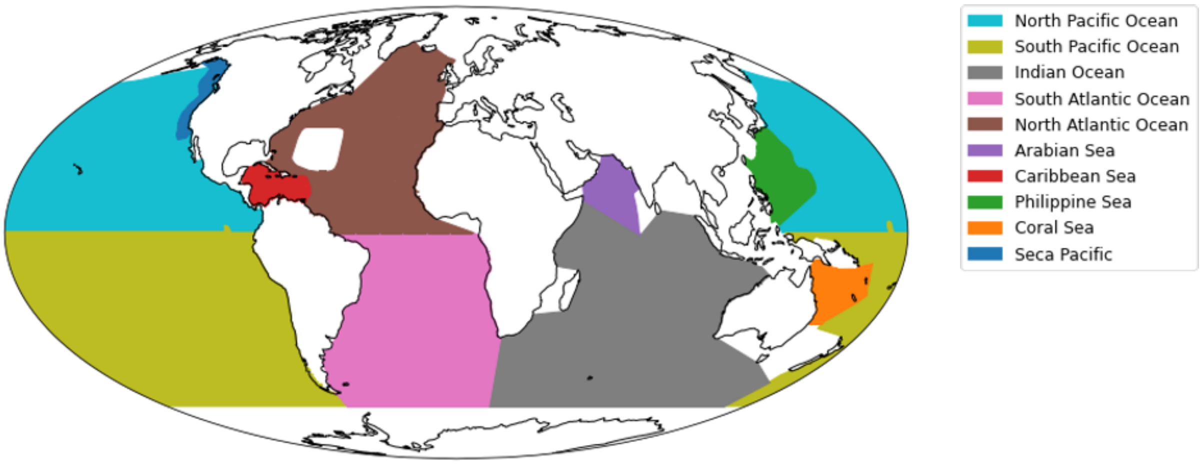

Fig. S7. Ocean basin regions used for the classification in Figure 2.

Model	IoU	# tracks / tile
FPN	0.41	3.0
ResUNet	0.39	3.5
**ResUNet augmented**	0.53	2.7

Table S1. Test Intersection-over-Union scores and average shiptrack counts for three distinct machine learning models. The average number of shiptracks detected in the hand-logged test dataset was 2.2. The model used for inference to generate the results presented in the manuscript is highlighted in bold.
